# Secondary prevention of UV-induced skin cancer: development and pilot testing of an educational patient counseling approach for individual sun protection as standard procedure of patient care

**DOI:** 10.1007/s00420-020-01532-7

**Published:** 2020-03-11

**Authors:** Michaela Ludewig, Marc Rocholl, Swen Malte John, Annika Wilke

**Affiliations:** 1 Department of Dermatology, Environmental Medicine and Health Theory, Institute for Health Research and Education, University of Osnabrück, Osnabrück, Germany; 2 Institute for Interdisciplinary Prevention and Rehabilitation at the University of Osnabrück, Osnabrück, Germany

**Keywords:** Sun protection, Skin cancer prevention, Germany, Individual patient counseling, Outdoor worker

## Abstract

**Objective:**

To outline the development and pilot testing of a patient counseling approach for individual sun protection for patients in outdoor professions diagnosed with squamous cell carcinoma or multiple actinic keratosis due to solar UV radiation. This is a secondary prevention measure as part of the standard procedure of patient care by the respective statutory accident insurance.

**Methods:**

Results of guideline-based qualitative interviews with seven outdoor workers and a search of literature formed the basis for the counseling approach, which was compiled in a manual. Interdisciplinary experts (dermatologists and educators) reviewed and consented the final manual. The pilot testing was conducted in consecutive steps (N_1_ = 36 patients and N_1_ = 2 counselors; N_4_ = 12 patients and N_4_ = 6 counselors). The first two stages of the revised guideline ‘Criteria for Reporting the Development and Evaluation of Complex Interventions in healthcare (CReDECI 2)’ serve as background and structure for presenting the results.

**Results:**

The ‘counseling approach for individual sun protection (ILB: Individuelle Lichtschutz-Beratung)’ consists of seven general and eight add-on modules. These can be combined and tailored according to the patient’s individual needs. Interactive, educational components (e.g. haptic experiments) are crucial elements of the counseling approach.

**Conclusions:**

The special requirements of outdoor workers are hardly taken into consideration in Germany, even though the interest for an improved sun protection behavior of some occupational groups is high. The advantage of the presented approach is that the heterogeneous needs of patients can be specifically addressed with its various modules.

## Introduction

Non-melanoma skin cancer (NMSC) is globally the most frequent cancer in fair skinned populations. Incidences are rising (Lomas et al. 2012). The causal relationship between UVR and skin cancer is established beyond doubt (Armstrong and Kricker 2001; World Health Organization (WHO) 2017; World Health Organization, International Agency for Research on Cancer 1992). In this paper, we focus on NMSC—especially squamous cell carcinoma (SCC) and actinic keratosis (AK = in situ SCC), located in sun-exposed areas of the skin (Sober and Burstein 1995). Since outdoor workers are particularly exposed to UVR, they are at a high risk of experiencing adverse health effects caused by solar UVR (Bauer et al. 2011; Schmitt et al. 2011). The estimated 14.5 million outdoor workers in the EU are exposed to solar UVR at least 75% of their working time. This concerns in particular farmers, gardeners, park maintenance staff, postmen, newspaper deliverers, physical education teachers, road builders, carpenters, and childcare workers (European Agency for Safety and Health at Work 2009). In Germany, 2–3 million people work in outdoor professions (Diepgen et al. 2012, 2015).

Knuschke et al. (2014) reported a two to three times higher UVR exposure for outdoor workers than for indoor workers. Therefore, sun protection to prevent the harmful health effects of UVR is a major concern for outdoor workers. However, various studies point out that the knowledge about sun protection measures of the (predominantly male) outdoor workers in Germany is very low (Unverricht and Knuschke 2007; Hault et al. 2016). High-risk groups are not yet aware of the issue (Zink et al. 2017). In this context, Bauer et al. (2015) reported a low compliance of outdoor workers with regard to the use of sun protection measures. Trakatelli et al. (2016) furthermore found a lower health literacy for outdoor workers. In their systematic review, Reinau et al. (2013) and Ziehfreund et al. (2019) identified a number of factors to be considered as typical barriers for individual sun protection behavior, namely forgetfulness, time-consuming application, sticky consistency of sunscreens, and feeling of heat when wearing hats and long-sleeved shirts. Nonetheless, Zink et al. (2017) demonstrated that farmers, roofers, and gardeners display a rather high interest in the topic of ‘sun protection.’ Furthermore, Duffy et al. (2018) showed in their study that knowledge about sun protection and availability of sunscreen can have positive effects for operating engineers.

In 2015, the federal government with consent of the federal council officially recognized ‘squamous cell carcinoma or multiple actinic keratosis due to natural UV radiation’ as an occupational disease. Thus, the German statutory accident insurance bodies can subsequently recognize these forms of skin cancer as an occupational disease (Berufskrankheiten-Verordnung (BKV) 1997; Zink et al. 2017). In view of the occupational disease and the large number of suspected cases (9.905 in 2018), preventive measures in terms of primary and secondary prevention are urgently needed for the UVR-exposed workers and patients already suffering from actinic damage as defined by the above-mentioned occupational disease.

Outdoor workers are, as mentioned above, a heterogeneous group. As Wittlich (2017) demonstrated, this also implies that the standard erythema dose (SED)^1^ can vary considerably across occupational groups. Taking the construction industry as an example, the highest annual exposure values are achieved by the duct builders (581 SED). In comparison, operators of construction machinery have lower exposure value (39 SED). In between are carpenters (474 SED), concrete workers (457 SED) and scaffolders (293 SED) (Wittlich 2017). Against this background, an individual counselling approach seems to be useful. Currently, no approach to counsel patients, i.e. affected workers, with NMSC is being applied in Germany. Hence, this paper aims to describe the development of a patient counseling approach for individual sun protection designed to address this specific at-risk population and thus fill the existing gap in occupational health services. In the following, we report the implementation and formative evaluation in accordance to ‘Criteria for Reporting the Development and Evaluation of Complex Interventions in healthcare (CReDECI 2)’ (Möhler et al. 2015).

## Materials and methods

Individual patient counseling for sun protection is a complex intervention in the field of disease prevention and health promotion. For that reason, we considered the Medical Research Council guidance for developing and evaluation complex interventions (Craig et al. 2008, 2013) as theoretical basis for our work because a systematic approach for the development of the intervention is needed.

### Literature research

First, we reviewed medical literature, international statements, and German guidelines for skin cancer prevention and sun protection (Elsner et al. 2007; Deutsche Krebsgesellschaft, Deutsche Krebshilfe, AWMF (Leitlinienprogramm Onkologie) 2014; International Commission on Non-Ionizing Radiation Protection 2010). Through the analysis of the material with a specific focus on sun protection measures, we identified the evidence base for our intervention. The leading question was as follows: What do outdoor workers need to know and to learn about the topic ‘sun protection’ and related areas in order to practice and implement an improved sun protection behavior? (see Table 1). We focused on contents for the counseling approach (e.g. clothes for sun protection) and especially on educational elements, (e.g. experiments), which illustrate these contents.

**Table 1:** Overview: exemplary recommendations for sun protection, ‘TOP principle’ (Bauer et al. 2015; Deutsche Krebsgesellschaft, Deutsche Krebshilfe, AWMF (Leitlinienprogramm Onkologie) 2014; Elsner et al. 2007; International Commission on Non-Ionizing Radiation Protection 2010)

*T*	*Technical measures*
1	Using shadow (e.g. solar sails) as far as possible
2	Seeking shade from the sun during breaks (e.g. for breakfast)
*O*	*Organizational measures*
3	Avoiding sun at midday (11–15 o’clock)
4	Appropriate work scheduling (e.g. starting in the early morning)
5	Providing sun protection measures (like sunscreens)
*P*	*Personal measures*
6	Wearing textiles for sun protection (shirt and trousers)
7	Wearing headgear (e.g. broad brimmed hat)
8	Wearing sunglasses
9	Using high sun protection factor (SPF) and correct application (e.g. cream amount)

Moreover, PubMed, Psyndex, and ERIC were searched for intervention studies in this field. The search strategies were integrated from the sections skin cancer, sun protection, outdoor work*, prevention, and intervention. Publications in English and German were included. The results of this process, in particular with regard to the implementation of a new behavior, were used to develop intervention strategies for the counseling approach.

### Guided, problem-centered qualitative interviews

We then needed to clarify the relevance for the recipients (Meisert 2013). Craig et al. (2008) recommended primary research activities to identify interests and needs of the target group. Therefore, guided, problem-centered qualitative interviews were conducted. The interviewees were seven male outdoor workers from different professional branches. In terms of sun protection, Rocholl et al. (2019) showed that the target group's attitudes were quite heterogeneous. This finding was also supported by Nahar et al. (2013) as they pointed out that older outdoor workers and people in outdoor professions with a longer work experience protect themselves better against UVR. Moreover, Zink et al. (2017) found that a low risk perception of skin cancer is associated with an insufficient use of protective measures. For this reason, the counseling approach should be tailored to every patient´s individual needs. Another crucial element identified, which has to be taken into account when developing the counseling approach, are the barriers perceived by outdoor workers, such as lack of time for applying sunscreen. The interview results will be reported in detail elsewhere (Rocholl et al. 2019).

### Aim and structure of the counseling approach and manual

The counseling approach is a face-to-face, short-term intervention with the objective of encouraging competences and strengthening the patient’s skills. Information and knowledge transfer are a salient part of this approach (Schaeffer and Petermann 2011). Processes of change should be enhanced with scientifically validated methods. Therefore, the counseling approach complies with the definition of professional health (care) advice (Krane 2011).

In light of all collected data, a first manual was drafted. For this purpose, we used the recommendations of the German Center for Patient Education (Küffner and Reusch 2014). These cross-indication formulated recommendations refer exclusively to group programs. Thus, we additionally considered the recommendations made by ‘EULAR’ (European League Against Rheumatism) (Reusch et al. 2017). While these criteria are exclusively limited to rheumatology, they nevertheless refer to online programs and standardized, individual counseling services (Reusch et al. 2017). A common element of both recommendations is the concept of shared decision making in training for patients.

### Multidisciplinary expert review and consensus

In a second step, a multidisciplinary expert review of the counseling approach was performed with experts (occupational dermatologists experienced in assessing patients with occupational skin cancer, also with regard to the approval of occupational diseases, as well as health educators experienced in developing, implementing, conducting, and evaluating concepts for patient education courses in the field of occupational skin diseases) in our clinical institution, where interdisciplinary inpatient and outpatient programs have been developed and implemented, comprising both dermatological patient management and health education interventions (Skudlik et al. 2008; Wilke et al. 2012). One specific inpatient prevention measure with a strong interdisciplinary approach is the so-called 'tertiary individual prevention' (Skudlik et al. 2008). It was established for patients with severe work-related hand eczema. The interdisciplinary team is experienced at conceiving health educational programs, implementing patient education programs, and conducting prospective studies (Brans et al. 2016).

In two expert meetings with different participants, contents and material were discussed based on central questions. The discussion was logged and the protocol was analyzed in a qualitative way:

Dermatologists (*N* = 5) and educators (*N* = 3) participated in the first meeting. The main focus was on the medical background of the occupational disease (development, progress, and therapy) and the practicability of the recommendations. The aim was to test the content of the educational material developed for the face-to-face counseling and to verify whether the material is appropriate in medical profoundness.

At the second expert meeting, educators from our two centers^2^ (*N* = 6 and *N* = 4) were requested to assess the educational material in terms of clarity, easy to read, and comprehensiveness for the target group. Furthermore, we gathered the experts’ opinions on how to best to reach out to the patients and on how to organize the counseling. Opportunities for interdisciplinary collaboration were discussed in both expert meetings. The results of this review process were analyzed qualitatively, and the manual and materials were revised accordingly.

### Pilot testing and implementation

The pilot testing was the part of the formative evaluation process which consisted of several steps. The contents and exercises were developed according to the needs of patients with occupational skin diseases. Patients with hand eczema and patients suffering from actinic damage are the both seeking medical care at our institute. Therefore, we chose this population for pilot testing the counseling approach. For this purpose, patients suffering from work-related hand eczema were included in the development for research economic reasons, in the first two steps. Since sun protection is relevant for these persons in their leisure time, this approach can also be justified methodically in order to test the comprehensibility of individual counseling elements and sequences.

One of the advantages of this procedure was that modifications and adaptations of content and sequences could be tested repeatedly in small steps and soon, based on the questions and comments of the participating patients. Hence, both women and men were initially included and patients in employment and self-employed persons were taken into account. The age range was from 20 to 60 years. First, two health educators tested single sequences of the approach, which are cohesive in content in a series of skin protection seminars (Skudlik et al. 2008) (e.g. regarding the development of skin cancer). This first step included six seminars with six participants each over a period of six weeks (N_1_ = 36 patients and N_1_ = 2 counselors). This was followed by a revision and adaption of the contents.

In a next step, the counseling approach was tested in inpatients and outpatients working in outdoor professions (e.g. construction workers) diagnosed with work-related hand eczema (Skudlik et al. 2008; Wilke et al. 2012) (N_2_ = 12 patients and N_2_ = 2 counselors). In this phase, the educational elements (e.g. experiments about sun protection) were evaluated, revised, and adapted accordingly.

In the next step, the entire intervention was tested (N_3_ = 2 patients and N_3_ = 1 counselor). In Appendix Fig. 3, the procedure of the intervention is described. The patients were outdoor workers diagnosed with multiple AK, recruited by the responsible accident insurance. Both workers were male aged 60 years, in employment, and 65 years but retired. The full-length face-to-face patient counseling for sun protection lasted for two hours.

Thereafter, the concept was implemented by several counselors in two locations. These health counselors have a pedagogical academic background, are experienced in patient education programs, and hold a train-the-trainer course certificate for quality assurance (Küffner and Reusch 2014). Prior to the course, they received a one-day training as well as literature and other material to prepare for the course. In addition, a contact person was available to reply to any questions of the counselors. To ensure that the counselor can conduct the intervention properly, a report was written for each counseling and reviewed by a second trained counselor. The following phase at both locations included twelve patients (current or retired outdoor workers with NMSC) and six health educationalists (N_4_ = 12 patients and N_4_ = 6 counselors). Ten men (aged 56 to 81 years) were included. Seven of these persons were retired at the time of the counseling, two were in employment, and one person was looking for work. In addition, two women (in employment, aged 27 and 54 years) were included. At their own request, four male patients took part in the intervention together with their wives.

Furthermore, a semi-structured interview sheet with 10 open-ended questions was constructed (Ahmed et al. 2016). It was used for generating qualitative data for the formative evaluation process. In a telephone survey (telephone interviews) 6–8 weeks after the intervention which was conducted by 'their' respective counselor, all participants were asked to give a feedback. The questions focused on the content of the counseling, the framework conditions, and the patient´s experience with our recommendations in his daily life and at the workplace. The interview guide has different sections with a total of 10 questions:

*Opening*

1. How are you?
  *Assessment of the intervention by the patient*
2. How did you like the UV protection counseling?
3. What changes do you think should be implemented for future counseling?
4. Which topics were of importance to you?
5. Which topics were less important?
6. Which topics did you miss?
  *Patient experiences*
7. Which recommendations could you implement after the sun protection counseling?
8. Did you have any problems with the implementation of the UV protection measures?
9. What could you not realize?
  *Assessment of the organization*
10. Did you feel satisfied with the organization of the measures?

*End of conversation*

Agreement with the patient with the information that further questions are possible by e-mail as well as by telephone.

The answers were recorded in writing by the interviewer.

## Results

The patient counseling approach for individual sun protection is a complex intervention that includes several components (Campbell et al. 2000). Reporting about the development and the pilot testing is necessary to ensure transparency (e.g. with regard to the theoretical basis) and a successful transfer into practice. Thus, the first two stages of the revised guideline ‘CReDECI 2’ by Möhler et al. (2015) provided the framework and structure for presenting the results below in this chapter.^3^

### First stage: development

#### Description of the intervention’s underlying theoretical basis

We developed an individual face-to-face intervention. The underlying theoretical basis is the result of our literature research. Selected aspects are described as follows:

In their systematic review of cross-sectional and interventional studies, Reinau et al. (2013) were able to show that sun safety education in outdoor occupational settings has proven effective in fostering outdoor workers’ sun protection habits. Eid and Mallach (2009) identified five predictors for sun protection measures:

- perceived risk for skin cancer,
- (dis-)advantages of different protection measures,
- knowledge about skin cancer and adequate protection measures,
- self-perceived capabilities in terms of conducting the protection behavior,
- social support.

Craciun et al. (2012) applied the health action process approach (HAPA) (Schwarzer 2008) in their study considering the application of sunscreen. Therefore, we chose the HAPA model as underlying health psychological theory to develop a sun protection intervention. The HAPA model distinguishes between ‘non-intenders’ who form an intention in the initial motivation phase as well as ‘intenders’ and ‘actors’ in the volitional stage (Schwarzer 2008). Ideally, patient counseling should be tailored to the particular needs of the patients who may be at different stages and have different 'mindsets' (Schwarzer 2008) of behavioral change. There is, for example, the need for ‘non-intenders’ to learn that sun protection has positive outcomes. ‘Some level of risk communication’ (Schwarzer 2008) might be necessary: In our case, this contains profound knowledge about skin cancer resulting from cumulative actinic damage of the skin without getting sunburned. If the participant belongs to the group of the ‘intenders,’ counseling targets supporting his action and coping planning since forming specific action and coping plans is essential for changing behavior according to the HAPA model. If the participant is an ‘actor,’ relapse prevention skills can be focused on if necessary (Schwarzer 2008). In this context, the previous behavior can be strengthened, success can be made visible, and potential distractors can be dismissed to prevent relapse.

For the development of the counseling approach, we used the recommendations of the German Center for Patient Education (Küffner and Reusch 2014). Elements, which were chosen to change attitudes and to motivate the patients, are given as follows:

- encouraging to create an intention,
- formulating and adjusting specific goals (e.g. formulating smart goals—*s*pecific, *m*easurable, *a*chievable, *r*ealistic/*r*elevant, and *t*imed (Bovend'Eerdt et al. 2009)—with focus on the own behavior),
- using specific counseling techniques.

#### Description of all intervention components, including the reasons for their selection as well as their aims/essential functions

The counseling approach consists of seven basic and eight add-on modules (see Table 2). Each patient completes all basic modules. Based on his prior knowledge, the patient can decide with the counselor which modules are more or less discussed in detail. The modules can be combined individually, depending on the needs (state of knowledge for the counseling (Faller et al. 2011) of the patient. Each module is approximately 10 min long. The total length of the intervention amounts to 2 h (see Appendix Fig. 3).

**Table 2:** Overview: basic and add-on modules of the approach for individual patient counseling

Basic module	Topic
Module 1	Start: myths about ‘sun protection’
Module 2	Basics: structure of the skin
Module 3	Basics: natural UVR
Module 4	Basics: non-melanoma skin cancer (NMSC)—‘actinic keratosis (AK) and squamous cell carcinoma (SCC)’
Module 5	Prevention: technical, organizational, and personal measures
Module 6	Glove protection counseling
Module 7	Individual action and coping planning

Add-on module	Topic
Module 8	Occupational disease No. 5103
Module 9	Sun protection after organ transplantation/immunosuppression
Module 10	Coping with heat
Module 11	How to deal with sunburn
Module 12	Mechanism of action of sunscreen products
Module 13	Eye exposure to UVR
Module 14	Tanning beds
Module 15	Sun protection for (grand-)children

Add-on modules were created as a result of the pilot testing for patients with special needs (e.g. immune-suppressed outdoor worker). Organ transplanted recipients have an increased risk for the occurrence of NMSC because of their inevitable immunosuppression (Berg and Otley 2002). Terhorst et al. (2009) showed that recreational sun exposure is one risk factor for developing NMSC in this group.

**Fig. 1:**
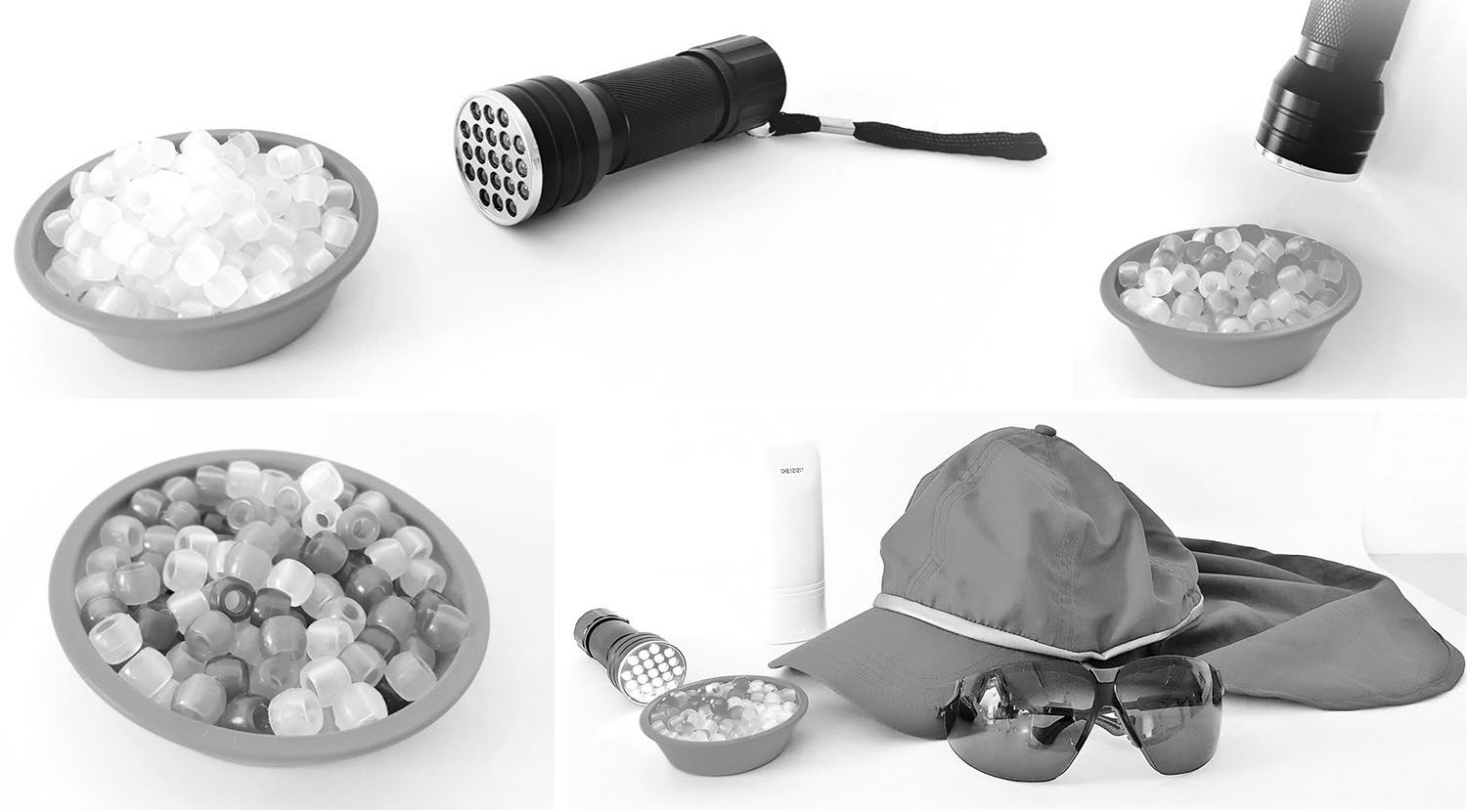
UV beads (colorless pearls that change color under UVR) used as educational material for UV experiments

Depending on the patient’s needs, the methods in the modules are chosen by the counselor. The teaching aims and methods for module 5 are described in Table 3 by way of example. Interactive, educational components (e.g. illustrations) are main elements of all modules to ensure a methodical, patient-oriented alignment (Küffner and Reusch 2014). For instance, as shown in Fig. 1, the so-called UV beads are being used to make invisible ultra violet radiation visible with a UVR emitting flashlight. Thanks to these colorless pearls that change color in the presence of UVR, we can show the protective effect of personal measures.

**Table 3:** Example for one of the basic modules—first stage: development

Module 5	Teaching aims	Methods
Prevention	5.1 The participant conceives the idea of the UV Index and is capable of using it in daily live	Working out the main points from a graphic in a dialog between patient and counselor
	5.2 The participant knows which factors influence the intensity of UVR	Brainstorming to identify these factors in personal daily life (work and leisure time) of the participant, followed by a discussion of results
	5.3 The participant knows different skin types and classifies her/his own skin type	Exercise to classify own skin type—focus on the duration of time an individual's skin can protect itself from UVR exposure
	5.4 The participant knows technical and organizational sun protection measures	Discussion about possibilities to use this measure at the workplace and in his/her leisure time (e.g. with different questioning techniques)
	5.5 The participant knows personal sun protection measures and she/he can apply them at his/her workplace and in his/her leisure time appropriately	Experiments on personal sun protection measures (e.g. with UVR sensitive pearls)

Another relevant educational aspect, which has proved successful in the pilot testing, is the involvement of spouses in the process of behavior change (Küffner and Reusch 2014). In the telephone interviews, the participants mentioned that they supported the transfer of the acquired knowledge into daily practice.

### Exemplary counseling situation

Within the framework of the ILB, a 59-year-old insured person participates, who is employed as a lifeguard in an indoor swimming pool as well as in an outdoor pool during the summer months. At first, an anamnesis of the insured person's workplace is elaborated, which serves as the basis for the counseling process. Working and break times, work processes, as well as already applied or available UV protection measures are recorded on the basis of the TOP principle (see Table 1).

Afterward, the basics listed in Table 2 (e.g. actinic keratosis and squamous cell carcinoma) are elaborated with the participant. For this purpose, skin cancer models, among others, are used. The participant can observe the skin changes both optically and haptically. This enables the outdoor worker to acquire knowledge that can be necessary for his own risk awareness. UV protection measures relating to the lifeguard's workplace will be discussed later. Personal possibilities for improvement of the protection behavior are worked out together (e.g. use of sunshades or awnings outdoors, applying the right amount of sunscreen, or wearing long-sleeved clothes). Possible barriers (e.g. lack of time to apply sunscreen, sweating in long-sleeved clothes) are identified and possible solutions are discussed (e.g. use of break times for putting on sunscreen). There are various materials available for the removal of these barriers (e.g. long-sleeved clothing, sunglasses, and various sun protection products for testing) and these are used as required.

It should be emphasized that the counseling sessions vary from person to person. In addition to the different professions, this is also related to the skills that the clients need.

#### Illustration of any intended interactions between different components

**Fig. 2:**
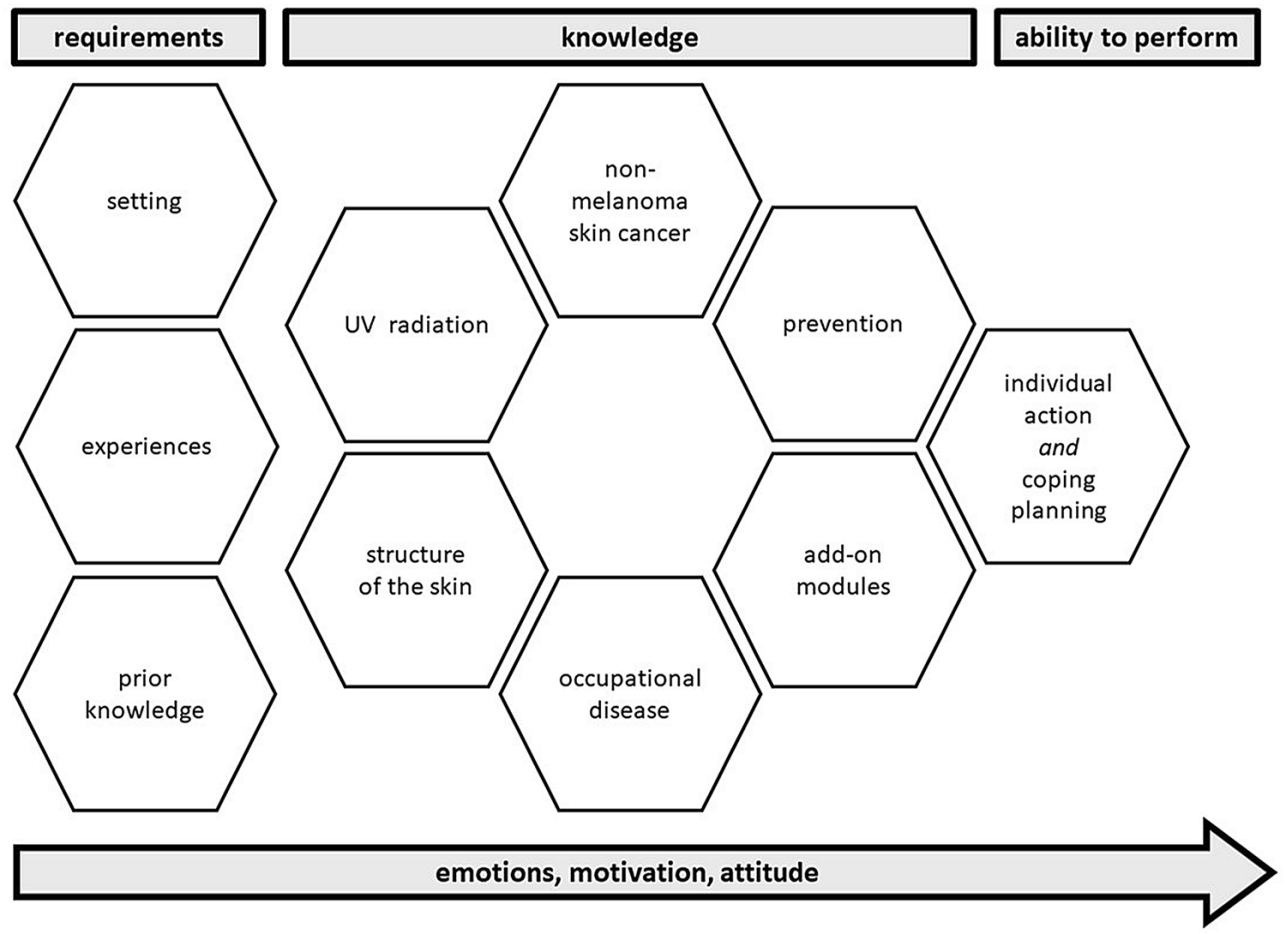
Components of a patient counseling approach for individual sun protection

The objective of the intervention is to enable patients to implement adequate sun protection measures (see Table 1). Based on this objective, Fig. 2 visualizes according to the guideline CReDECI 2 the relationship between the different components of the intervention:

*Requirements* The intervention is based on the requirements and needs of each participant, including his knowledge on sun protection prior to the intervention, which is very heterogeneous, and very much depends on the setting (e.g. workplace), where the participant lives, and on his experiences (e.g. social support in terms of sun protection from his colleagues). These requirements were included in the counseling approach.

*Knowledge* Based on the above, the modules thus have different priorities. Some of them emphasize knowledge acquisition (e.g. UVR) in order to create a common state of knowledge between participant and counselor. It furthermore aims at raising the outdoor worker’s awareness for the harmful aspects of UVR. Other modules focus on practical implementation and motivational strategies (e.g. experiments).

*Ability to perform* One module focuses on 'individual action planning,' which is most relevant since it directly impacts the transfer of the newly acquired behavior into daily life. To that end, the outdoor worker develops his own sun protection plan during the counseling by including his individual strategies and addressing possibilities for optimizing his behavior. This plan is to overcome barriers occurring in everyday life (Sniehotta et al. 2005).

#### Description and consideration of the context characteristics in intervention modelling

The counseling approach was developed under the following framework conditions at different levels:

*Macro level* In accordance with Article 3 of the German Ordinance on Occupational Diseases, the German Social Accident Insurance must ensure the prevention of occupational diseases with all appropriate means. Against this background, in a European consensus report, leading scientists call for steps to increase knowledge about the risk of AK and NMSC among occupational workers to empower and change behavior among these groups (John et al. 2016).

*Meso level* Due to the high incidences in Germany, for the last two decades, our institute (Skudlik 2015) has been developing educational approaches and interdisciplinary programs for occupational skin diseases, including work-related hand eczema (Wilke et al. 2015; Sonsmann et al. 2015), in cooperation with the German social accident insurances.

*Micro level* The team that has been developing this face-to-face counseling approach has acquired over the years a vast knowledge on structures for interdisciplinary work, including from other occupational diseases (Skudlik et al. 2008; Wilke et al. 2012).

### Second stage: feasibility and piloting

#### Description of the pilot testing and its impact on the definite intervention

The pilot testing was done in a series of steps and is (as well as its impact) described above (see paragraph 2 “Materials and methods”). Based on responses of the participants in the telephone survey and counselors in the team meetings, the final version of the counseling approach was confirmed.

The pilot testing indicated that the counseling approach was well received and accepted by the outdoor workers and the counselors. Both groups rated the intervention with the school grades good or very good. In the telephone interviews, the individual focus of the intervention was often mentioned as a particularly positive feature of the counseling approach. Patients indicated that they were satisfied with the procedure and the content and did not see any further requirements regarding other topics. Furthermore, the participants reported the following actual behavior changes in the telephone interviews:

- installation of a sunshade for selected activities;
- watching of the UV index for planning the UV protection behavior;
- use of a headgear with neck protection;
- use of sunglasses;
- use of long-sleeved clothing;
- use of protective gloves to protect hands from solar UV radiation;
- routine developed for applying the cream (e.g. in the morning after brushing teeth);
- an increase in the amount of sunscreen used.

The counselors were also contented with the intervention. They considered the different methods of working with the patients by means of a person-centered approach, the support provided in case of questions, as well as the feedback on their counseling reports to be quite helpful. This feedback confirmed that this intervention addresses common unmet needs, and revealed that participants enjoyed and valued the counseling as they acquired specific skills for managing sun protection. Our results indicate that the developed and tested counseling approach can be delivered by staff associated to an occupational dermatology and health education center. This is one possibility of implementing this intervention in skin cancer prevention for outdoor workers.

## Discussion

This paper describes the development and pilot testing of a patient counseling approach for individual sun protection in outdoor professions. To the best of our knowledge, there is no individual, face-to-face intervention for outdoor workers with NMSC in Germany. Details about possible effects and the success factors can thus only be derived from literature from other countries. Existing German campaigns, interventions, or information primarily address children and their parents (Arbeitsgemeinschaft Dermatologische Prävention (ADP) 2018a, b; Bundesamt für Strahlenschutz 2017; Deutsche Gesetzliche Unfallversicherung (DGUV) 2014) and target much less the general population, such as campaigns 'Slip! Slop! Slap!' in Australia do (Marks 1990). We were able to take various aspects of these programs into account when developing our intervention. These were especially integrated in the sun protection modules for leisure time.

Furthermore, the special requirements of outdoor workers are hardly taken into consideration in Germany, even though the interest for an improved sun protection behavior of some occupational groups is high (Zink et al. 2017). Regardless of the profession, the participants of our pilot testing confirmed this assumption and emphasized their interest in the topic.

In addition, we found two important aspects during the pilot testing, which can influence the intervention negatively if not borne in mind: heterogeneous education levels in outdoor workers (Zink et al. 2017) and a low health literacy (Trakatelli et al. 2016) underline an obvious demand for simple messages. Moreover, one of the advantages of a personal counseling (as reported by our patients in the telephone interview) is the possibility of direct feedback, e.g. no individual questions raised by illustrations or explanations. Other interventions, such as mobile apps, usually do not offer this possibility. The effectiveness of apps (e.g. the use of mobile apps that show the photoaging effects of solar UVR on the participant's face) has so far been tested mainly on younger target groups (e.g. students) (Brinker et al 2017, 2018a, b). Little is known about the use in older people (Brinker et al 2018a, b). However, disadvantage of personal counseling compared to group training is the lack of exchange with persons who also suffer from the disease.

Furthermore, it should be considered that individual skin health attitudes are influenced by various factors, including not only public education campaigns and mass media as well as family and friends (Haluza et al. 2015) but also colleagues in working environments (Nahar et al. 2013). For that reason, we also invited the accompanying persons of our participants, such as spouses, to take part in the counseling, if the participant agreed with it. The feedback of all involved persons was very positive because the interaction and the discussion atmosphere changed in a constructive way (e.g. more questions were asked) and the accompanying persons were able to support the behavior change in everyday life. According to Schwarzer (1999), the HAPA model comprises barriers and resources. Spouses could be a personal resource in this context. In the future, further research could focus on this aspect.

Additionally, the ILB participants reported on the general conditions at their working environments (e.g. availability of technical protective measures) as well as about colleagues who, for example, influence UV protection behavior through social reactions to the use of neck protection. If such aspects were considered in the course of prevention, an increase in the effectiveness of the measures would be conceivable. For this reason, prevention options in the setting ‘workplace’ should be examined as already recommended by Schilling et al. (2018).

Some limitations have to be discussed: Single content sequences and counseling elements were tested with patients suffering from hand eczema. Some of them were significantly younger than the patients typically suffering from occupational disease no. 5103. However, a younger age may be associated with a lower risk behavior (Rocholl et al. 2019). Therefore, this group of persons was appropriate for testing of contents and elements for the so-called ‘non-intenders’ (Schwarzer 1999). In spite of the encouraging results from the pilot testing of this intervention, it was developed and primarily delivered by an experienced team of health educators. Yet, more effort is needed to establish the intervention in other settings where counselors (e.g. medical assistants) may not have such practical health educational training but their capacities should be used, since they have access to patients in the doctor's offices. The formative evaluation aimed at identifying strengths and weaknesses of the counseling approach (Scriven 1996), rather than delivering any detailed health-related outcomes. These data will be considered in a summative evaluation. The degree of evidence of the counseling effectiveness was based on Agency for Healthcare Research and Quality corresponds to level IV (Blümle et al. 2009). One strength of this approach is the patient-oriented design of the counseling. The contents can be worked out on the basis of the individual risk factors (e.g. skin type) of the outdoor workers as Seite et al. (2017) suggested. Furthermore, it should be considered that patient education can directly influence proximal outcomes such as knowledge, motivation, change in attitude, and gaining skills as well as ability to perform (Küffner and Reusch 2014). Whether behavioral changes will be implemented successfully also depends on the conditions at different workplaces (Hammond et al. 2008). Further strengths of the intervention are the systematic development in accordance with the evidence-based recommendations, the consideration of the target group ‘outdoor worker’ who has special challenges due to his/her job, and the consideration of the HAPA model as a psychological basis.

There is indeed a lack of interventions for improved sun protection behavior of outdoor workers. In future projects, prevention should thus focus particularly on the following:

### Primary prevention

Patient education trainings for primary prevention in this field are needed for instance including such interventions in vocational schools. In addition, multipliers (e.g. occupational physicians or occupational health and safety practitioner) should be qualified for teaching at outdoor workplaces. Our participants expressed the wish to conduct primary prevention education programs at their workplace together with colleagues. Another aspect could be to make use of specific communication channels used by the most-at-risk groups (including youth, men, and individuals belonging to a lower socio-economic class or having a lower education level), like Seite et al. (2017) claimed for educational campaigns as well.

### Secondary prevention

Patient care can be further improved by developing additional, more precise modules for different occupational groups (e.g. farming or construction). In this way, the transfer of the counseling approach into practice could be made easier, since these modules could, for example, concretely describe possibilities of implementation regarding various sun protection measures in different working environments.

Moreover, future research should especially focus on summative evaluation using validated tools with larger sample sizes that examine, for example, patient-reported health outcomes. It is also advisable to systematically explore both proximal (e.g. behavior) and distal outcomes (e.g. symptoms) or to investigate the constructs of the HAPA model and to gain further in-depth insight into possible intervention effects of the counseling approach.

## Conclusions

It has been shown that solar radiation can have both positive and negative effects on humans depending on exposure time. It is important that outdoor worker know about their increased sensitivity caused by intensive occupational exposure to UVR, so that they may adapt their behavior accordingly. To maximize health outcomes, individuals should be educated—especially high-risk groups. Pilot testing indicated that the counseling approach was well-received and accepted by outdoor worker as well as counselors. This feedback confirmed that this intervention addresses common unmet needs and revealed that participants valued the counseling. Our pilot testing indicate that this counseling approach can be delivered by existing staff associated with an occupational dermatology and health education center.

In Germany, campaigns and interventions for UVR protection mostly focused on leisure time and corresponding activities. Almost all address the general population—especially children and their parents. Now, an approach for individual counseling in terms of specific occupational UVR-protection for outdoor worker is available and can be included in other prevention strategies of NMSC contexts. Nevertheless, effectiveness in terms of behavioral change of our counseling approach has to be proven in future studies.

## Figures and Tables

**Fig. 3:**
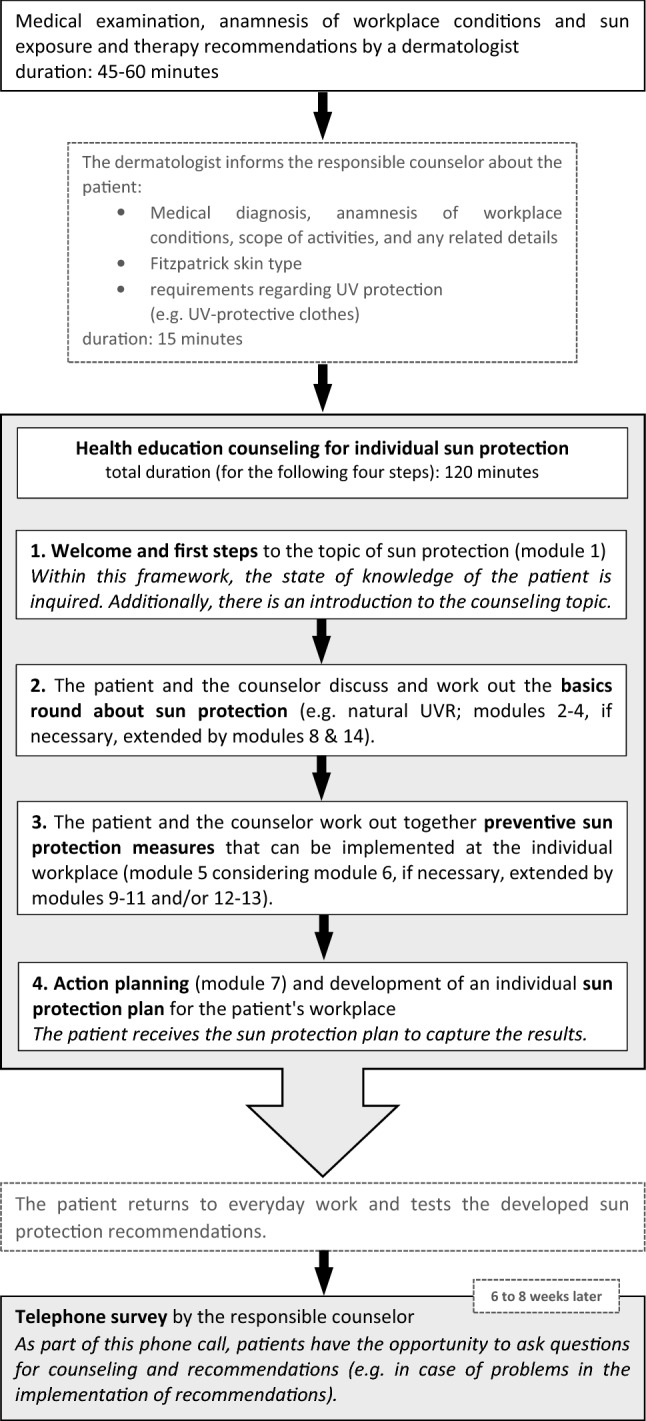
Procedure of the counseling approach for individual sun protection

## Data Availability

The data used to support the findings of this analysis are included within this article.

## Appendix 1 Procedure of the counseling approach for individual sun protection

See Fig. 3.

## Footnotes

1 1 SED may cause sunburn in skin type I (Wittlich 2017).

2 The centers of our institute are located in Osnabrück and Hamburg.

3 The results part is divided into two main parts according to the guideline: first stage development (criteria 1–4) and second stage: feasibility and piloting (criterion 5). The criteria are the subheadings and structure these two areas.
